# A Lack of Correlation between Brain-Derived Neurotrophic Factor Serum Level and Verbal Memory Performance in Healthy Polish Population

**DOI:** 10.3389/fncir.2016.00039

**Published:** 2016-05-23

**Authors:** Monika Wilkosc, Anita Markowska, Ludmila Zajac-Lamparska, Maria Skibinska, Agnieszka Szalkowska, Aleksander Araszkiewicz

**Affiliations:** ^1^Institute of Psychology, Kazimierz Wielki UniversityBydgoszcz, Poland; ^2^Department of Psychiatry Nursing, Collegium Medicum in Bydgoszcz, Nicolaus Copernicus UniversityTorun, Poland; ^3^Psychiatry Genetics Unit, Poznan University of Medical SciencesPoznan, Poland; ^4^Department of Psychiatry, Collegium Medicum in Bydgoszcz, Nicolaus Copernicus UniversityTorun, Poland

**Keywords:** serum mBDNF, serum proBDNF, Rey Auditory-Verbal Learning Test (RAVLT), verbal memory, healthy population

## Abstract

Brain derived neurotrophic factor (BDNF) is considered to be connected with memory and learning through the processes of long term synaptic potentiation and synaptic plasticity. The aim of the study was to examine the relationship between precursor BDNF (proBNDF) and mature BDNF (mBDNF) serum levels and performance on Rey Auditory-Verbal Learning Test (RAVLT) in 150 healthy volunteers. In addition, we have verified the relationships between serum concentration of both forms of BDNF and RAVLT with sociodemographic and lifestyle factors.We found no strong evidence for the correlation of proBDNF and mBDNF serum levels with performance on RAVLT in healthy Polish population in early and middle adulthood. We observed the mBDNF serum concentration to be higher in women compared with men. Moreover, we revealed higher mBDNF level to be connected with lower body mass index (BMI). In turn, the results of RAVLT correlated with sociodemographic and lifestyle factors, such as: age, education, gender, BMI and smoking.

## Introduction

One of the most crucial cognitive functions is memory. Through the processes of encoding, storage and retrieval of information, an individual collects and recall experience and knowledge underlying its everyday functioning. The Rey Auditory Verbal Learning Test (RAVLT; Rey, 1964) is one of the most extensively used methods to evaluate the components of verbal memory. It allows to assess the capacity of immediate memory storage, abilities and strategies of learning new information, susceptibility to interference or ability to recall learned material after a delay (Strauss et al., 2006). RAVLT allows also the identification and differentiation of irregularities in the mechanisms of auditory-verbal memory in different disorders (Greenaway et al., 2006; Johnsen and Asbjørnsen, 2008).

Over the years, evidence was given that the performance on RAVLT is influenced by sociodemographic factors. The results of studies found the age in adults to be strongly negatively correlated with verbal memory efficacy measured by RAVLT (Bolla-Wilson and Bleecker, 1986; Crossen and Wiens, 1994; Ferreira Correia and Campagna Osorio, 2014). The research has also revealed a positive correlation between performance on RAVLT and level of education (Ferreira Correia and Campagna Osorio, 2014; Bezdicek et al., 2014). However, according to Schmidt, (1996) this correlation should be considered inconsistent since it varies from low to moderate. Furthermore, in gender-related studies females were more often reported to perform RAVLT better than men (Schmidt, 1996; Gale et al., 2007; Bezdicek et al., 2014).

In addition, the relation between lifestyle factors, including smoking and body mass index (BMI) with RAVLT performance, was also tested. According to Starr et al. (2007) smoking influence negatively the RAVLT outcome, although Paul et al. (2006) found no relation between these variables. However RAVLT results varied depending on whether the test conducted on smokers was preceded by a period of abstinence from nicotine (Soar et al., 2008). There was also a relationship between BMI and parameters of RAVLT which was independent of age (Gunstad et al., 2006).

During the performance of memory tasks, including RAVLT, multiple brain regions forming a neurofunctional memory system become active. Medial temporal lobe, particularly hippocampus, plays a central role in the processes of declarative memory for different materials, including verbal-auditory one (Zola-Morgan and Squire, 1990; Zeineh et al., 2003; Nagel et al., 2004). The hippocampal volume loss correlated with significant worsening of memory processes in different disorders (Braak and Braak, 1991; Woon et al., 2010), as well as in the course of normal aging (De Toledo-Morrell et al., 2000; Van Der Flier et al., 2005).

Brain-derived neurotrophic factor (BDNF) is recognized as one of the important correlates of atrophy of hippocampus and related memory deficits. A wide array of research concerned relationships of its functional polymorphism Val66Met both with the hippocampal volume (Pezawas et al., 2004; Szeszko et al., 2005; Bueller et al., 2006) and memory (Kambeitz et al., 2012) in healthy and clinical populations. Recently, the role of BDNF protein concentration in serum, blood plasma or cerebrospinal fluid is increasingly tested. BDNF protein belongs to the family of neurotrophins. It is responsible for the growth, differentiation and survival of neurons (Bath and Lee, 2006). It has been shown to play an important role in induction of long term synaptic potentiation (Pang et al., 2004) and synaptic plasticity (Bekinschtein et al., 2007) that underlie memory processes. BDNF gene is located on chromosome 11p13 (Maisonpierre et al., 1991). It has a complex structure and regulation of expression. It consists of 9 exons, of which eight 5’ exons are non-coding and only exon 9 encoded BDNF protein. This gene has 9 promoters, 3 alternative polyadenylation signals and alternative splicing points. Protein in the form of pre-proBDNF, proteolytically cleaved to proBDNF with a mass of 32 kDa, is formed on a matrix of all transcripts. ProBDNF can undergo further proteolytic cleavage by enzymes such as furin and pro-converting enzymes and is secreted outside the cells as mature BDNF (mBDNF) with a mass of 14 kDa. Both proBDNF and mBDNF are packaged in secretory vesicles and secreted constitutively and dependently on activity of neurons. Both forms of protein are biologically active. Binding of proBDNF with receptor p75NTR activates the processes of apoptosis, while mBDNF, as homodimers, upon binding to the receptor TrkB has a trophic effect (Cunha et al., 2010). BDNF expression occurs in CNS structures, such as cerebral cortex, cerebellum and limbic system structures, mainly hippocampus, amygdala and olfactory bulb (Ernfors et al., 1990; Huang and Reichardt, 2001). In healthy people, the level of circulating BDNF increases with age until it reaches an adult level in the third decade of life, and begins to decrease in late adulthood (Lommatzsch et al., 2005; Katoh-Semba et al., 2007; Li et al., 2009; Erickson et al., 2010). Some studies conducted on older adults show a positive relationship between the level of circulating BDNF and the volume of hippocampus or functioning of memory processes (Gunstad et al., 2008; Komulainen et al., 2008; Li et al., 2009; Erickson et al., 2010). Gunstad et al. (2008) and Li et al. (2009) postulated that decreasing level of mBDNF may constitute one of the important factors responsible for cognitive decline in older adults. Also Komulainen et al. (2008) observed the relationship between mBDNF level and cognitive functions, including verbal memory. There are also data which showed a negative correlation (Niitsu et al., 2011) or no correlation (O’Bryant et al., 2011; Driscoll et al., 2012; Kim et al., 2015) between serum BDNF and memory functions in healthy subjects. Previous reports have also found that sex may covariate with BDNF concentration (Karege et al., 2002; Lommatzsch et al., 2005).

Other factors being examined in the context of relations with the level of BDNF include: BMI, smoking and physical activity. Lommatzsch et al. (2005) have shown that the BDNF plasma level in healthy people decreases significantly with an increase of body weight. Araya et al. (2008) confirmed the relationship between weight and BDNF plasma concentration level in overweight and obese people who have gone on a diet. BDNF concentration increased after 3 months of the diet. In turn, Zhang et al. (2008) revealed the relation between the level of BDNF and BMI in the group of women with schizophrenia.

In case of relations between BDNF and nicotine dependency, it was found that non-smokers have higher levels of BDNF in serum compared with smokers (Kim et al., 2007; Bhang et al., 2010). They have also showed increasing levels of BDNF following smoking cessation. On the other hand, Suriyaprom et al. (2013) observed nicotine-induced upregulation of BDNF in Thai males, while Zhang et al. (2010) in schizophrenic patients.

The results of the studies showed also an elevation in the BDNF level after physical exercises (Ferris et al., 2007; Tang et al., 2008). It was postulated that the magnitude of this increase depends on exercise intensity (Ferris et al., 2007). However, the BDNF response to acute training seems to be short-lasting (Knaepen et al., 2010). Moreover, it seems that erobic exercises have the largest effect on increase of BDNF concentration, while strength training have no influence (Huang et al., 2014). In addition, Winter et al. (2007) showed the relationship between the increased BDNF serum level after physical exercise and cognitive functioning, including verbal memory and learning.

The aim of the study was to analyze the association between proBDNF and mBDNF serum levels and performance on RAVLT in 150 healthy volunteers. In addition, we verified the relationship between proBDNF, mBDNF serum levels, RAVLT with sociodemographic and lifestyle factors.

## Materials and Methods

The study included 152 healthy volunteers (76 females and 76 males) aged 18–60 years (*M* = 38.362; *SD* = 11.571) enrolled on the basis of detailed clinical interview without history of any psychiatric disorder, or serious somatic illnesses. All subjects were in intellectual norm measured by polish version of raven’s progressive matrices. We also controlled BMI, smoking (number of cigarettes per day) and physical activity (number of days of minimum half an hour physical activity per month). Any medical treatment was an exclusion criterion. all subjects were caucasians of polish origin. We provided the written informed consent. Bioethics Committee of Collegium Medicum in Bydgoszcz, Nicolaus Copernicus University in Torun approved the study.

Ten milliliters (10 ml) of venous blood was withdrawn into anticoagulant-free tubes between 7.30 and 9.30 a.m. to minimize effects of a circadian rhythm of BDNF concentrations. it was done after overnight fast. After 1 h incubation serum was separated by centrifugation, aliquoted and stored at −70 until analysis. Elisa analyses were performed using DuoSet ELISA Development Kits (R&D Systems): mBDNF (DY248) and proBDNF (DY3175) according to manufacturer’s instructions, with minor modifications. Plates were blocked for 3 h in reagent diluents (1% BSA/PBS). Serum samples for BDNF analysis were diluted 1:100 in reagent diluents prior to assay, to fit the linear range of standard curve. Serum samples for proBDNF were run undiluted. Plates were incubated with 100 μl of serum samples overnight at room temperature. All samples and standards were run in duplicates. Standard curves ranged: 1500–24.4 pg/ml. mBDNF and proBDNF inter- and intra-assay variability was <5% cv.

To assess verbal memory we used Polish version of RAVLT translated from English (Lezak et al., 2004). The test consists of two lists containing 15 nouns: list A (main) and list B (distractive). Words from list A were read by a researcher in fixed order once before each of five free recall trials (RAVLT_A1-A5). After completion of fifth one, the words from list B were read once. A participant was asked to recall the words from list B (RAVLT_B) and then the words from list A (recall after distraction—RAVLT_A6). After 20 min, a participant was asked to recall the words from list a again (delayed recall—RAVLT_A7; Lezak et al., 2004). The number of words recalled in each trial (RAVLT_A1-A7 and RAVLT_B) was evaluated. Attention and short-term memory are needed throughout all trials, however the number of words recalled at RAVLT_A1 and RAVLT_B are considered the best indicators of short-term memory capacity. Learning curve is formed by a total number of words recalled in five attempts (RAVLT_A1-A5), the final acquisition level measured by RAVLT_5 and delayed recall assessed by RAVLT_A7 (Ferreira Correia and Campagna Osorio, 2014).

Statistical analysis was performed in four steps, using STATISTICA 12.0 and IBM SPSS Statistics 21 along with PROCESS macro, to assess:

1. Relationship of RAVLT results with demographic and lifestyle factors;
2. Correlation between RAVLT performance and the serum levels of proBDNF and mBDNF;
3. Relationship of serum proBDNF and mBDNF levels with demographic and lifestyle factors;
4. Association of RAVLT results with sociodemographic and lifestyle variables in interaction with serum proBDNF and mBDNF levels (Moderated Multiple Regression).

## Results

The characteristics of the subjects in regards to sociodemographic and lifestyle variables are presented in Table 1.

**Table 1:** **Characteristics of the participants**.

Variable	Whole group (*N* = 152) M (SD)	Females (*N* = 76) M (SD)	Males (*N* = 76) M (SD)
Age (years)	38.362 (11.571)	38.697 (11.379)	38.026 (11.825)
Education (years)	15.783 (3.303)	16.632 (2.925)	14.934 (3.458)
Weight (kilograms)	76.283 (16.149)	66.671 (10.409)	85.895 (15.148)
Height (meteres)	1.727 (0.083)	1.667 (0.051)	1.786 (0.064)
BMI	25.449 (4.250)	23.996 (3.631)	26.901 (4.347)
Smoking (cigarettes per day)	3.874 (8.492)	1.960 (5.740)	5.813 (10.257)
Physical activity (days per month)	4.553 (7.009)	3.000 (5.381)	6.105 (8.066)

### RAVLT Results and Age, Sex, BMI, Smoking, Physical Activity

The analysis of relationship between RAVLT performance and age, sex, BMI, smoking, physical activity showed that the results of RAVLT depended on all considered sociodemographic and lifestyle variables.

Student-*t* test revealed that verbal memory in all RAVLT parameters was significantly better in women than in men. The results are presented in Table 2.

**Table 2:** **The comparison of Rey Auditory-Verbal Learning Test (RAVLT) results in females and males**.

	Females (*N* = 76)		Males (*N* = 76)	
RAVLT	*M*	*SD*	*M*	*SD*	*t*-test	*p*-value
A1	8.000	2.046	7.210	2.446	2.158	0.032*
A2	11.092	2.161	10.158	2.509	2.459	0.015*
A3	12.553	2.055	11.553	2.397	2.761	0.006**
A4	13.592	1.722	12.447	2.036	3.743	0.0003***
A5	13.934	1.526	12.882	1.986	3.663	0.0003***
B	8.053	2.534	7.066	2.168	2.579	0.011*
A6	12.303	2.613	11.013	3.101	2.772	0.006**
A7	12.658	2.392	11.066	2.990	3.625	0.0004***
A1–A5	59.171	8.314	54.250	9.945	3.309	0.001**

*p < 0.05; **p < 0.01; ***p < 0.001.

In turn, on the basis of the correlation analysis, we observed that the better results in all components of RAVLT correlated with younger age and longer time of schooling. Among lifestyle variables, we found the negative correlations of the results of all RAVLT parameters with BMI and the number of cigarettes smoked daily. We observed no relation between RAVLT results and physical activity (Table 3).

**Table 3:** **Pearson’s correlations of RAVLT results with sociodemographic and lifestyle variables (*N* = 152)**.

	Age		Education		BMI		Smoking		Physical activity	
RAVLT	*r*	*p*	*r*	*p*	*r*	*p*	*r*	*p*	*r*	*p*
A1	−0.358	<0.001***	0.355	<0.001***	−0.355	<0.001***	−0.211	0.009**	0.135	0.096
A2	−0.342	<0.001***	0.337	<0.001***	−0.266	0.001**	−0.264	0.001**	0.093	0.252
A3	−0.401	<0.001***	0.325	<0.001***	−0.290	<0.001***	−0.235	0.004**	0.120	0.141
A4	−0.362	<0.001***	0.385	<0.001***	−0.349	<0.001***	−0.249	0.002**	0.034	0.679
A5	−0.372	<0.001***	0.246	<0.001***	−0.330	<0.001***	−0.245	0.002**	0.008	0.921
B	−0.438	<0.001***	0.323	<0.001***	−0.350	<0.001***	−0.219	0.007**	0.087	0.287
A6	−0.345	<0.001***	0.288	<0.001***	−0.303	<0.001***	−0.338	<0.001***	0.127	0.120
A7	−0.391	<0.001***	0.369	<0.001***	−0.369	<0.001***	−0.329	<0.001***	0.113	0.164
A1–A5	−0.416	<0.001***	0.377	<0.001***	−0.377	<0.001***	−0.273	0.001**	0.094	0.251

*p < 0.05; **p < 0.01; ***p < 0.001.

### BDNF Serum Levels and Age, Sex, BMI, Smoking, Physical Activity

The assays of mBDNF levels in serum were indicated in 140 persons (72 women and 68 men) aged *M* = 38.300 *SD* = 11.573 (whole group), *M* = 38.630 *SD* = 11.449 (females) and *M* = 37.960 *SD* = 11.778 (males). The assays of proBDNF levels were indicated in 90 persons (55 females and 45 males) aged *M* = 36.167 *SD* = 11.249 (whole group), *M* = 37.127 *SD* = 11.351 (females), *M* = 34.657 *SD* = 11.080 (males). For the rest of the group BDNF concentrations were at undetectable level. The mean values of mBDNF and proBDNF serum concentration in studied group are presented in Table 4.

**Table 4:** **mature BDNF (mBDNF) and precursor BDNF (proBNDF) serum level in studied group**.

Serum concentration (pg/ml)	Whole group *M (SD)*	Females *M (SD)*	Males *M (SD)*
mBDNF	29156.320	30737.230	27482.420
	(7490.505)	(8316.215)	(6130.626)
proBDNF	466.640	452.310	489.150
	(728.648)	(682.903)	(805.135)

In order to examine gender-related differences, we carried out Student-t test. We found the mBDNF concentration to be higher in women than in men (*t* = 2.623, *p* = 0.010).

Further, we analyzed Pearson’s correlation of mBDNF and proBDNF serum levels with the other sociodemographic and lifestyle variables. The results indicated the existence of statistically significant interchangeability between the BMI index and mBDNF concentration. Higher value of BMI was connected with lower mBDNF serum concentration (*r* = −0.186, *p* = 0.028).

### BDNF Serum Levels and RAVLT Results

To address our main question about relation between pro-BDNF and mBDNF serum levels and RAVLT, we conducted *r*-Pearson test. We found no significant correlations between these variables (Table 5).

**Table 5:** **Correlations between proBDNF and mBDNF and results of RAVLT**.

	proBDNF			mBDNF		
RAVLT	*N*	Pearson’s *r*	*p-value	*N*	Pearson’s *r*	*p-value
A1	90	−0.033	0.757	140	0.123	0.148
A2	90	0.024	0.823	140	0.093	0.274
A3	90	0.026	0.807	140	0.138	0.104
A4	90	0.042	0.694	140	0.164	0.058
A5	90	0.050	0.639	140	0.149	0.079
B	90	0.029	0.789	140	0.149	0.080
A6	90	0.040	0.707	140	0.051	0.546
A7	90	0.034	0.751	140	0.094	0.267
A1–A5	90	0.022	0.835	140	0.148	0.081

*p < 0.05.

### RAVLT Results and Sociodemographic and Lifestyle Variables in Interaction with Serum ProBDNF and mBDNF

Moreover, we applied a regression factor analysis in which, for particular RAVLT parameters, the sociodemographic and lifestyle factors, as well as proBDNF or mBDNF were included as independent variables. Three interactive dependences turned out to be significant. the results of RAVLT_A2 were explained by interaction between the age and proBDNF concentration: *F*_(1,86)_ = 4.067, *p* = 0.047, $\eta_{p}^{2}$ = 0.045. Moreover, RAVLT_A5 were explained by interaction between the number of cigarettes smoked daily and the mBDNF concentration: *F*_(1,135)_ = 4.028, *p* = 0.047, $\eta_{p}^{2}$ = 0.029. Finally, RAVLT_A6 were explained by interaction between physical activity and concentration of mBDNF: *F*_(1,136)_ = 5.510, *p* = 0.020, $\eta_{p}^{2}$ = 0.039.

For conditional effects modeling we used moderation model 1 in “PROCESS” for SPSS (Hayes, 2012). For the analysis as depending variable (*Y*), independent variable (*X*) and moderator (*M*) the following were included accordingly:

1. *Y*—result of RAVLT_A2, *X*—age, *M*—proBDNF;
2. *Y*—result of RAVLT_A5, *X*—number of cigarettes smoked daily, *M*—mBDNF;
3. *Y*—result of RAVLT_A6, *X*—number of days per month in which physical activity is performed, *M*—mBDNF.

**Figure 1:**
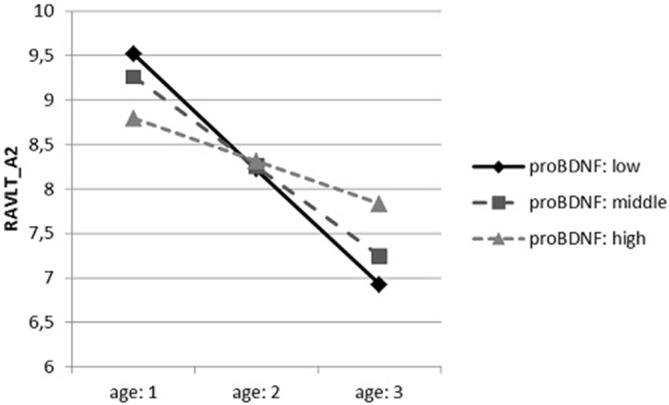
**The dependence of the results of Rey Auditory-Verbal Learning Test (RAVLT)_A2 on the age, moderated by precursor BDNF (proBNDF) concentration**.

In case of the first interactive dependences (Figure 1), conditional effects indicated a significant relation between the age and verbal memory measured by RAVLT_A2. The higher the age was, the worse performance we observed. However, it was revealed only in participants with the lowest proBDNF concentration (*t* = −5.123, *p* < 0.001) and the average one (*t* = −4.416, *p* < 0.001), but it was not significant in persons with the highest concentration of proBDNF (*t* = −1.302, *p* = 0.196).

**Figure 2:**
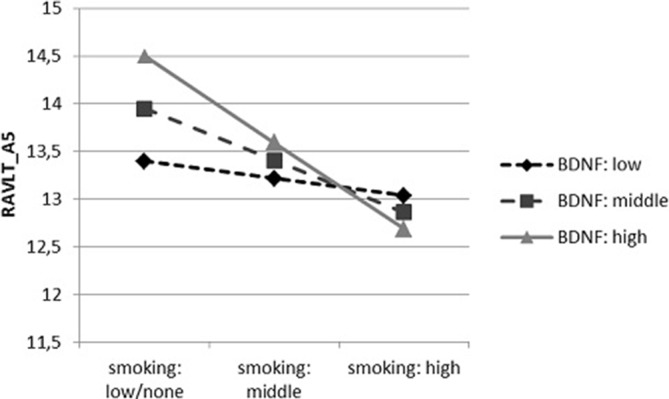
**The dependence of the results of RAVLT_A5 on the number of cigarettes smoked per day, moderated by mature BDNF (mBDNF) concentration**.

In case of the second interactive dependence (Figure 2), we found that negative relation between the number of cigarettes smoked daily and the results in RAVLT_A5 remained significant only in persons with average (*t* = −3.474, *p* < 0.001) and high (*t* = −3.415, *p* < 0.001) concentration of mBDNF, and there was no relation in participants with low mBDNF concentration (*t* = −0.861, *p* = 0.390).

**Figure 3:**
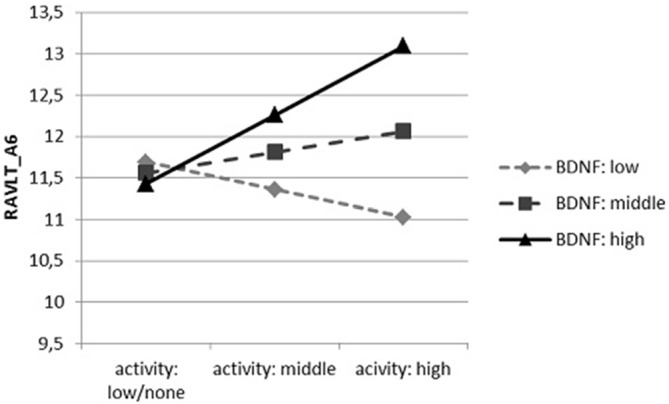
**The dependence of the results of RAVLT_A6 on the physical activity (number of days in a month in which physical activity is performed), moderated by proBDNF concentration**.

In accordance with the third interactive dependence (Figure 3), a significant positive relation between physical activity and the results of RAVLT_A6 occurred only in participants with the highest mBDNF concentration (*t* = 2.613, *p* = 0.010), while in case of persons with the average concentration we observed a statistical tendency with an analogous character (*t* = 1.773, *p* = 0.078). No significant relationship in participants with the lowest concentration of mBDNF was found (*t* = −0.425, *p* = 0.672).

## Discussion

The main focus of the present study was to analyze the relationship between verbal memory and serum BDNF levels, in precursor and mature forms. In literature, results of several studies indicated a positive relation between BDNF level and cognitive functions in healthy persons (Gunstad et al., 2008; Erickson et al., 2010), including verbal memory and learning processes (Komulainen et al., 2008; Li et al., 2009). Moreover, the results of mediation analysis between serum BDNF level, hippocampus volume and performance on a spatial memory test (Erickson et al., 2010) implied that volume of the left hippocampus mediates the association between BDNF and spatial memory. Based on these reports we expected the higher mBDNF serum level to be correlated with better performance on RAVLT. However, correlation analysis showed no significant relationship between these variables. Obtained results are compliant with other studies (O’Bryant et al., 2011; Driscoll et al., 2012; Kim et al., 2015). All earlier positive findings on relation between BDNF concentration and cognitive performance were revealed in older populations. Our findings may suggest a little effect of circulating mBDNF on memory performance in healthy adults in early and middle adulthood, while its impact in late adulthood, especially in age-related cognitive decline or pathological states may be much greater. Also we may hypothesize that BDNF influence the spatial memory more than verbal one. Moreover, we obtained null results for proBDNF but there are no findings to refer to since we found no reports about its influence on cognition in healthy population.

We also examined the relation between serum concentration of both forms of BDNF and sociodemographic factors. We found that neither form of BDNF correlated with age. However, in our group we have not included participants over 65 years of age in whom the decrease of circulating BDNF appears to be most significant (Erickson et al., 2010). Also we found mBDNF level to be higher in females. This in agreement with previous reports that found gender-related differences in BDNF concentration (Karege et al., 2002; Lommatzsch et al., 2005; Shimada et al., 2014), also in association with verbal memory performance (Komulainen et al., 2008). However, the relation was found only in women, whereas the majority of them admitted to undergo hormonal therapy. There were endeavors to explain this difference with effects of estrogen which may be connected with BDNF expression (Scharfman and Maclusky, 2005). It is noteworthy, that none of female participants used hormonal therapy during the present study.

Additionally we assessed the relationship between concentration of both forms of BDNF and lifestyle factors, such as BMI, smoking and physical activity. We found lower BMI to be correlated with higher mBDNF serum level which is in accordance with earlier findings (Lommatzsch et al., 2005; Araya et al., 2008).

We also conducted additional analysis to assess the relationship between sociodemographic variables with verbal memory and learning. In agreement with previous studies (Bolla-Wilson and Bleecker, 1986; Crossen and Wiens, 1994; Schmidt, 1996; Gale et al., 2007; Salgado et al., 2011; Ferreira Correia and Campagna Osorio, 2014) we found age to be negatively, while years of education positively correlated with performance on all parameters of RAVLT (Ferreira Correia and Campagna Osorio, 2014; Bezdicek et al., 2014). Both correlations were strong (*p* < 0.001). It confirms that ageing in adult cohort progressively impairs the verbal memory, while educational level may play a protective role on mnemonic processes (Ferreira Correia and Campagna Osorio, 2014). Regarding age factor, Swanson (1999) found best performance on working memory task in the group of 24-year-old, while the weakening of memory function was observed even at the age of 35–40 years. In context of schooling period, it was suggested that higher education is connected with better engagement of phonological loop in auditory rehearsal which may result in higher scores on immediate recall measures (Teruya et al., 2009). Also, it can be assumed that higher educational level is achieved by individuals with superior learning abilities. Besides, in accordance with some earlier reports (Schmidt, 1996; Van Der Elst et al., 2005; Gale et al., 2007; Bezdicek et al., 2014), women outperformed man in all RAVLT parameters, corroborating the findings that women have better verbal abilities (Yonker et al., 2003). Additionally, interaction analysis showed that older age was connected with worse performance on RAVLT_A2. Nonetheless, it was observed in participants with the lowest and average concentration of proBDNF, while it was not significant in persons with the highest concentration of proBDNF. It may be assumed that high proBDNF serum level smoothed away the negative effect of age on verbal memory. However, the dependency was observed just in one of RAVLT immediate recall trials.

It also turned out that results of RAVLT were connected with lifestyle factors, such as BMI and smoking. Lower BMI was correlated with better results in all RAVLT domains. The majority of results from animal research (for review see, Kanoski and Davidson, 2011) indicated that a diet rich in saturated fats, which causes obesity, was related to deterioration of cognitive processes, including memory and learning. Gunstad et al. (2006) revealed the impairment of verbal memory and learning processes with obesity in comparison to the persons with normal weight or overweighed ones in all adult age groups. Moreover, they found no evidence of an interaction between BMI and age on memory which may suggest that the relationship between BMI and memory is independent of age. According to present study, smoking cigarettes had also an negative impact on verbal memory and learning measured by RAVLT. Similarly, results of Starr et al. (2007) indicated a worse level of immediate recall among smokers in comparison to non-smokers and former smokers. However, another research (Paul et al., 2006) did not show any differences in immediate recall between smokers and non-smokers, although older smokers performed worse in delayed recall than younger smokers and non-smokers. Soar et al. (2008) was examining RAVLT performance in persons addicted to nicotine before and directly after smoking. It turned out that smokers, who had not been smoking for 12 h, acquired new verbal material worse than smokers who were not deterred from smoking. After smoking, the level of learning in both groups was comparable. The negative relationship between number of cigarettes smoked per day and RAVLT results was also revealed in interaction analysis, however only in participants with average and high serum mBDNF concentration. This dependency was observed just for RAVLT_A5 trail which is predominantly connected with efficacy of acquisition of new material.

In correlation analysis we found no connection between physical activity and performance on RAVLT. However, interactive analysis revealed such relation in participants with high level of BDNF. This dependency was observed only in RAVLT_A6 trail which is mainly connected with resistance to interference. Earlier reports found that acute exercise temporarily elevates circulating BDNF level, upregulates BDNF synthesis and in consequence may induce neurotrophic and neuroprotective effects (Cotman et al., 2007).

There are some limitations of current study. First, our group did not consist of older adults in whom the BDNF influence on cognition seems to be the greatest. Second, we used one measure of memory, which additionally evaluated only auditory-verbal modality. However, in some of the previous studies in which a set of memory task was employed, the results also showed no association of BDNF level with memory performance (Kim et al., 2015). Third, we could control the lifestyle factors in more rigid way. We have not investigated i.e., the type of physical activity, neither its exact duration or frequency.

Overall, we have not uncovered the evidence for a relationship between serum level of proBDNF and mBDNF with verbal memory and learning. However, we revealed higher mBDNF in women. We also found mBDNF to be correlated with lower BMI. Additionally, we confirmed that age correlates negatively with results of RAVLT, while education is related positively. Moreover, we found negative relation of RAVLT performance with BMI and smoking.

Our results put additional light on still little understood role of circulating BDNF in cognition. Greater understanding of the influence of biomarkers as well as knowledge about the impact of sociodemographic and lifestyle factors on memory may be important in diagnosis, prevention and treatment of cognitive dysfunction.

## Author Contributions

Substantial contributions the conception or design of the work: MW, MS, AA. Substantial contributions to the acquisition of data: MW, AS. Substantial contributions to the analysis interpretation of data for the work: LZ-L, MS. Substantial contributions or interpretation of data for the work: MW, AM. Drafting the work or revising it critically for important intellectual content: MW, AM, LZ-L, MS, AS, AA. Final approval of the version to be published: MW, AM, LZ-L, MS, AS, AA. Agreement to be accountable for all aspects of the work in ensuring that questions related to the accuracy or integrity of any part of the work are appropriately investigated and resolved: MW, AM, LZ-L, MS, AS, AA.

## Funding

This work was supported by the National Science Center, granted on the basis of decision No. DEC-2011/01/B/HS6/00440.

## Conflict of Interest Statement

The authors declare that the research was conducted in the absence of any commercial or financial relationships that could be construed as a potential conflict of interest.
